# Review of control strategies for lower-limb exoskeletons to assist gait

**DOI:** 10.1186/s12984-021-00906-3

**Published:** 2021-07-27

**Authors:** Romain Baud, Ali Reza Manzoori, Auke Ijspeert, Mohamed Bouri

**Affiliations:** 1 Biorobotics laboratory (BioRob), EPFL, Lausanne, Switzerland; 2 Translational Neural Engineering Lab (TNE), EPFL, Geneva, Switzerland

**Keywords:** Exoskeleton, Lower-limb, Control, Review

## Abstract

**Background:**

Many lower-limb exoskeletons have been developed to assist gait, exhibiting a large range of control methods. The goal of this paper is to review and classify these control strategies, that determine how these devices interact with the user.

**Methods:**

In addition to covering the recent publications on the control of lower-limb exoskeletons for gait assistance, an effort has been made to review the controllers independently of the hardware and implementation aspects. The common 3-level structure (high, middle, and low levels) is first used to separate the continuous behavior (mid-level) from the implementation of position/torque control (low-level) and the detection of the terrain or user’s intention (high-level). Within these levels, different approaches (functional units) have been identified and combined to describe each considered controller.

**Results:**

291 references have been considered and sorted by the proposed classification. The methods identified in the high-level are manual user input, brain interfaces, or automatic mode detection based on the terrain or user’s movements. In the mid-level, the synchronization is most often based on manual triggers by the user, discrete events (followed by state machines or time-based progression), or continuous estimations using state variables. The desired action is determined based on position/torque profiles, model-based calculations, or other custom functions of the sensory signals. In the low-level, position or torque controllers are used to carry out the desired actions. In addition to a more detailed description of these methods, the variants of implementation within each one are also compared and discussed in the paper.

**Conclusions:**

By listing and comparing the features of the reviewed controllers, this work can help in understanding the numerous techniques found in the literature. The main identified trends are the use of pre-defined trajectories for full-mobilization and event-triggered (or adaptive-frequency-oscillator-synchronized) torque profiles for partial assistance. More recently, advanced methods to adapt the position/torque profiles online and automatically detect terrains or locomotion modes have become more common, but these are largely still limited to laboratory settings. An analysis of the possible underlying reasons of the identified trends is also carried out and opportunities for further studies are discussed.

**Supplementary Information:**

The online version contains supplementary material available at 10.1186/s12984-021-00906-3.

## Introduction

Powered lower-limb orthotic devices, also called powered exoskeletons, are often considered as tools in rehabilitation and the assistance of the human gait. A significant amount of research in different fields has been dedicated to developing and improving the performance of these devices, and there are many challenges in this area of research due to inherent requirements of portability and safe interaction with the user and the environment. One of the most important aspects for improving the performance of these devices is their control [1].

Currently, there are two main types of exoskeletons for gait assistance: the ones for full mobilization, and the ones for partial assistance. Full mobilization exoskeletons are designed to move the legs of people suffering from a severe loss of motor control or motor disorders, typically in people with spinal cord injury SCI. The actuators must have a high torque capability because they provide the entire torque required for the movement. Such devices are available commercially since 2011, when the ReWalk (ReWalk Robotics, Israel) was released on the market. They could be developed quickly because their control strategy can be simply position control over time. There is no need to collaborate with an existing voluntary movement of the legs, because there is none (or it is very weak) and thus the user’s legs are assumed to be passive. The start of the gait is often triggered by the upper body movements or buttons pressed by the fingers, which is simple to implement. These exoskeletons seem more successful because they dramatically improve the bipedal ambulation capability (from no gait at all to some slow gait).

Partial assistance devices are generally lighter, targeting various less severe handicaps. These could be the loss of stamina because of aging [2], the loss of strength or coordination because of incomplete spinal cord injury SCI, stroke, neurodegenerative diseases, etc. These devices can also assist the gait of healthy people, which can be useful for endurance augmentation purposes. This is more challenging because the device has to assist more than it is hindering its user, given the complex nature of the interaction with the user. People who can already walk independently also have higher expectations for the performance (e.g. higher gait speed). A major subcategory of partial assistance exoskeletons are the devices that are intended for rehabilitation purposes.^1^ Here, the ultimate purpose is to train the users to become independent of the assistance offered by the device. A fundamental distinction can thus be made between the desired outcomes of these exoskeletons versus the ones that are used to directly assist the mobility only when wearing the device. Actually, a training strategy for rehabilitation may consist in resisting the user movement [3]. Notwithstanding this difference in the end goals, there is a lot of commonality between the two applications in terms of the techniques used for control.

Several reviews already exist on different aspects of exoskeletons and gait assistance devices, but very few are focused on control. The two most exhaustive reviews of control strategies to date are the ones of Tucker et al. [4] and Yan et al. [5]. However, these are already 5 years old at the time of writing this paper, and many new developments deserve to be mentioned, since this field is evolving fast. More than 190 new publications addressing control strategies have been identified since the publication of the two previous reviews in 2015, and advancements have been made with new control methods and device designs, resulting in major performance improvements in terms of metrics such as metabolic cost reduction and capabilities such as crutch-less dynamic walking. The review of Tucker et al. is broad and considered both orthoses and prostheses. A “generalized control framework” was proposed with a 3-layer hierarchical controller, and also the environment, the user and the hardware of the device. But this review did not provide much detail on the mid-level layer of control. The article by Yan et al. focuses on the control of exoskeletons and orthoses, but it is mostly organized around the devices themselves, and how they are built (e.g. single/multi joints).

Some reviews have also been recently published on gait assistance devices [6–9], but none of them comprehensively address the control aspect. A recent review by Sawicki et al. [7] focused on comparing the results of partial assistance for the gait, and only considered the successful orthoses with respect to metabolic cost reduction. This excludes all the devices that did not undergo such testing and also full mobilization exoskeletons. Also in this article, few details are given on the details of the control part. A more broad review by Kalita et al. [8] studied the existing exoskeletons and orthoses in the literature, categorizing them according to joint structure, actuation and control strategy. Control strategies are roughly divided into 9 categories, each one only briefly explained without going into the details.

In this review, the various control approaches of gait assistance devices are thoroughly addressed, focusing on the lower-limb exoskeletons designed to enhance the locomotion of disabled or healthy people. Compared to the existing reviews, a stronger emphasis is placed on the control methods and separating them from the hardware and implementation details as much as possible. Based on the existing control methods in the literature, a modular classification framework consisting of 3 layers is proposed. The purpose of the framework is to enable describing all of the existing control strategies with the minimum number of functional elements. This paper also shortly reviews the metrics used to characterize the performance of these robots when worn by a user. However, the assessment of the performance of the cited controllers and their comparison are beyond the scope of this review.

## Assistive strategies

From the control perspective, the main challenge for gait assistance is to contribute to the intended movement, since the device cannot directly communicate with the wearer to clearly recognize the intention and collaborate effectively. Effective collaboration can be interpreted in different ways, depending on the context and application. In general, for partial assistance it would mean synergy in forces or torques between the user and the device, and for full mobilization it would be coordination between the movements of the exoskeleton and those of the user’s upper body. Many strategies are used to identify the user’s intent, and apply an appropriate torque or motion accordingly. In the rest of this section, the existing strategies will be reviewed and discussed. Before getting into the review of these strategies, the rationale behind the criteria that were used for screening the literature and the proposed classification method will be explained, and the methodological steps will be described.

### Methods

#### Scope and methodological steps

The main question to be addressed in this part is: what approaches have been used in the literature up to now for controlling lower-limb exoskeletons with the purpose of directly assisting the wearer’s gait? Target devices for the controllers in this review do not need to provide an improvement of the user’s health. Although the devices are typically anthropomorphic, exceptions also exist (such as [10–13]). The so-called “soft exoskeletons” (exosuits) are included too, even if these are not really stiff “skeletons”, but closer to “tendons and muscles”. The papers that do not deal directly with an exoskeleton, but suggest a sensing method that could be useful for them are included as well. As explained previously, many gait assistance devices are presented in the context of rehabilitation. In light of the similarities from the control perspective, we did not limit the scope of this review to a specific application; as long as the described controller is supposed to assist the user during gait, the method was included in this review regardless of the long-term goal.

This review aims to address *wearable* gait assisting exoskeletons, because they have the potential to be used for real-life applications out of the laboratory. However, the articles involving fixed-frame devices designed to explore such control strategies (e.g. LOPES [14], ALEX [15], the exoskeleton emulator of Collins et al. [16], etc.) are also included in this review. In addition, if at least part of the control strategy proposed for a fixed-frame rehabilitation device also assists the user’s gait and is applicable to assistive exoskeletons, it is included (for example [17]). The strength augmentation devices are excluded because they are not designed to enhance the walking mobility. The main consequence is that they are of no use for people affected with gait deficiencies, or healthy people willing to improve their ability to walk (higher speed and/or endurance) with no load. They also mainly focus on load lifting so the control strategies may be different, and may also involve upper limbs. The task of carrying a load while walking (e.g. [18]) is closer to the topic of this article, but such devices still do not assist in moving the user’s legs or relieve the user from the bodyweight. In addition, it makes comparing the performance even more difficult, because the assistance benefit depends on the amount of payload. However, a strength augmentation device that would enable its wearer to jump higher or run faster would have been included, but such reference could not be found. Similarly to the fixed-frame rehabilitation devices, a strength augmentation device can be still be included if at least part of the control strategy could be applicable to the assistance of the gait with no carried load (e.g. [19]). The inclusion and exclusion criteria used in the screening process of this review are summarized in Table 1.

**Table 1:** Summary of the inclusion and exclusion criteria used for screening the articles

Inclusion criteria	Exclusion criteria
$\bullet$ Includes description of controller(s) applicable to lower-limb exoskeletons with the purpose of helping wearer’s gait, or detection methods applicable to such controllers	$\bullet$ Describes a controller that is specific to other devices such as prosthetics, upper-limb exoskeletons, fixed-frame rehabilitation devices (such as Lokomat [326] and MotionMaker [327]) or portable devices that operate as external units rather than wearable robots (e.g. WalkTrainer [328]) or devices that were designed only for animals
$\bullet$ Date of publication: January 2000 to August 2020	$\bullet$ Describes a controller for devices that assist locomotion by using another movement than the natural movement of the human leg (such as rolling devices, jumping stilts in which the blade moves below the foot, jet-packs [329] and portable inertial devices [330, 331])
$\bullet$ Language of publication: English	$\bullet$ Describes a controller that is intended for assisting load-carrying or strength augmentation without significantly affecting the gait itself
$\bullet$ Type of publication: peer-reviewed journal or conference article, patent	$\bullet$ Describes a controller that is impossible to apply outside of a simulated environment
	$\bullet$ Does not give enough details about the control method to fully describe it (typically the case for papers reporting clinical trial outcomes)
	$\bullet$ Gives inconsistent information about the controller and/or the device
	$\bullet$ Only reviews control methods

Most of the publications were found using the following Google Scholar query:

robot* assist* control* (exoskele* OR orthosis) 

and a similar query on Scopus: “robot* assist*” “control*” AND ( exoskele* OR orthosis ) AND NOT ( “upper limb*” OR “upper-limb*” OR “hand exoskelet*” ) among the records published since January 2000 up to the end of August 2020. The references cited in the two previous review papers by Yan et al. [5] and Tucker et al. [4] were also included.

**Fig. 1:**
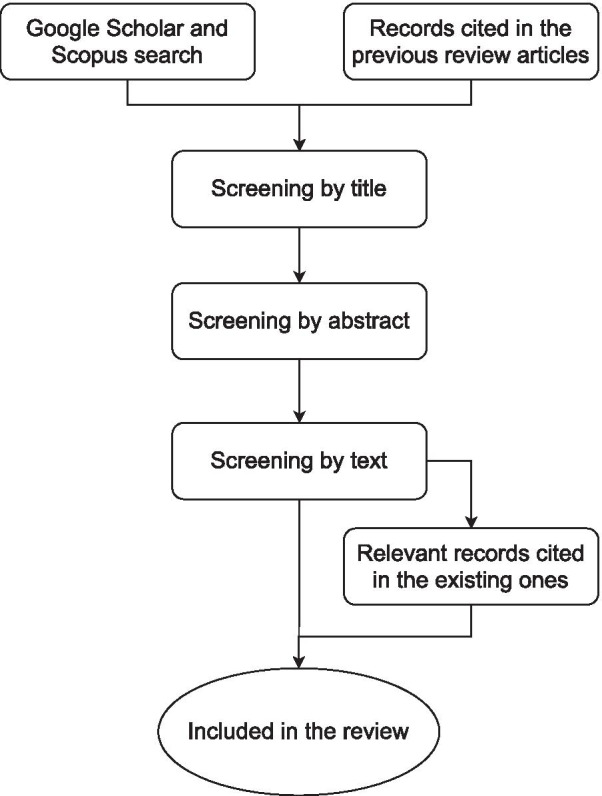
Flowchart of the methodology used for the search and screening process

First, the references were screened with the title, then the abstract, and finally the full-text to check if they fit the inclusion/exclusion criteria. Then, they were read entirely and entered in a database. The relevant articles cited by the ones already in the database were also added. A flowchart of the methodology is shown in Fig. 1. For each entry in the database, the following fields (as long as they were relevant/applicable) were entered: high-level control method, mid-level control method, low-level control method, type of actuator, short controller description, intended application, assisted joints, device name, and remarks.

#### Proposed classification

This review is centered on control strategies, being hardware-agnostic as much as possible. To be accurate enough in describing the different control strategies features, but with no redundancy in the descriptions, it was chosen to break the behavior into smaller functional units. Indeed, an initial assessment of the literature revealed that even among different control strategies, shared elements exist. Compared to describing each control strategy as an atomic entity, this classification method allows for reusing the same elements to represent several strategies.

**Fig. 2:**
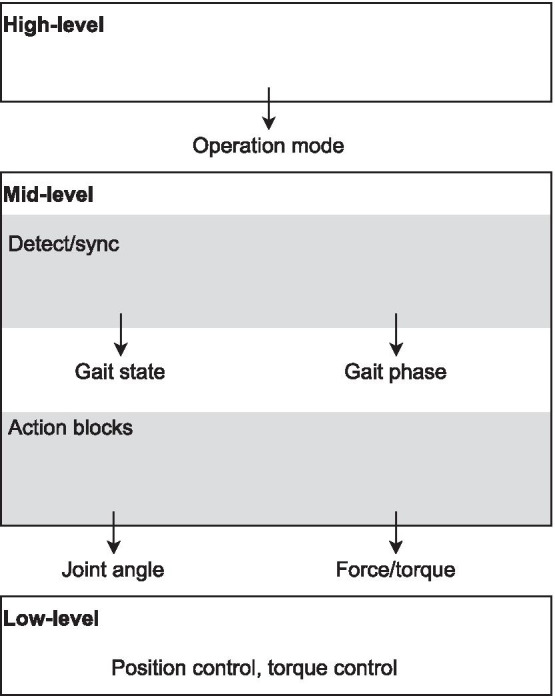
Simplified diagram of the proposed classification

The literature shows a considerable number of different controllers, with different structures, designs and actuation methods. However, the ultimate requirements in terms of performance and desired behavior are mostly similar. In an attempt to classify them, we will separate the controllers into smaller functional units that are comparable. Each functional unit can be used in several different combinations to form various controllers. Therefore, these functional units can be considered as the building “blocks” of the controllers. Based on their role in the hierarchy of the control system, all of these blocks can be classified into three categories: high-level, mid-level and low-level control (see Fig. 2). This hierarchical classification is similar to the one used in [4].

**Fig. 3:**
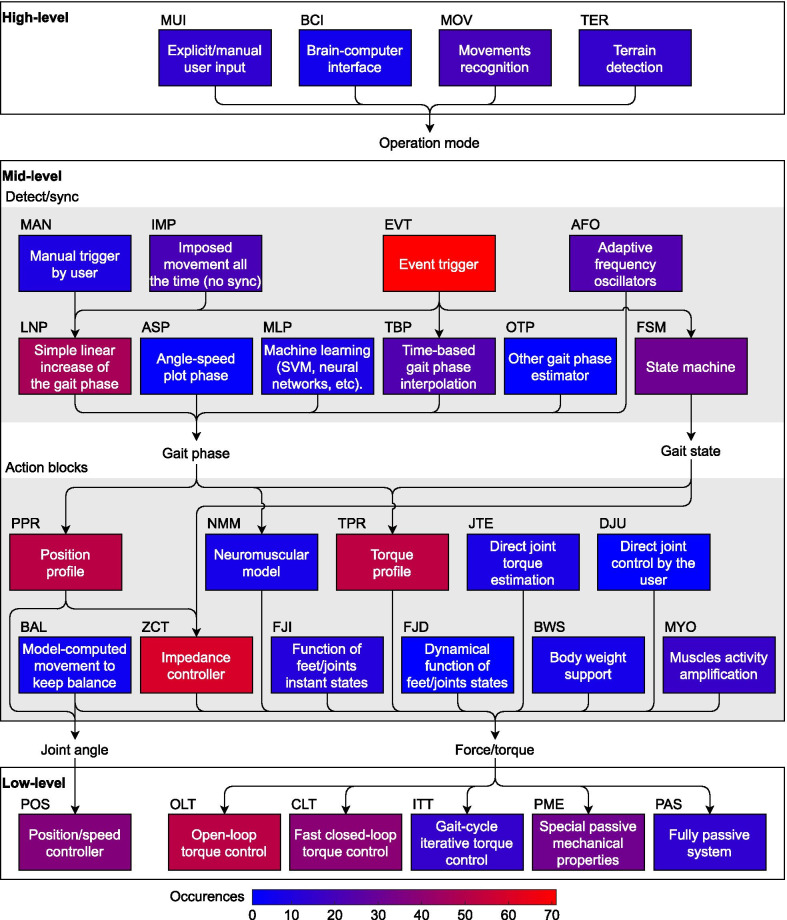
Block diagram of the proposed classification of the control strategies subparts. The idea of this classification is that any controller in the literature can be represented by a path that joins the used control blocks. The path does not have to start from the high-level layer, and may start directly in the mid-level. A controller can have several parallel paths if the controller combines several strategies at the same time, or successively during the gait. Connecting lines show the common paths identified in the literature. However, it should be noted that the lack of a line between two blocks does not mean they cannot be related. For instance, the outcome of the high-level layer, the “operation mode”, could affect any of the blocks of the middle-level, but it is not connected to them for the sake of readability

Within each level, various methods and approaches thus form the different blocks. Some of the blocks within the same level perform the same function (in terms of outputs) using different methods, while others have a dissimilar functionality. Hence, even though the blocks in different levels may be used together, they are not always compatible. All of these blocks are shown in Fig. 3 and will be explained in detail later in the paper. It should also be noted that the reviewed control strategies do not necessarily cover all the three levels, with most of the research being focused on mid-level control. This review will then focus on mid-level control mostly.

Our analysis of the high- and mid-level layers is also implementation-agnostic, which means it focuses on the external behavior of the device rather than the way to program it or make the hardware design. Most of the hardware-specific aspects will be separately discussed in the low-level layer.

The results obtained by all these controllers are not compared, because the target users are different (healthy, elderly, paraplegic, stroke, etc.), the tasks are different (walking, running, ascending stairs, etc.), and even for the same task, the experimental protocol is often different. Such comparison is possible, but only with a narrower scope. For example, the review of Sawicki et al. [7] focuses on the partial assistance for the gait, to decrease the metabolic cost of locomotion for healthy people.

### High-level control

The high-level control determines the general behavior of the exoskeleton. Exoskeletons can usually switch between several operating modes, depending on the desired type of activity, and the environment (e.g. walking on flat terrain, climbing stairs, and sit-to-stand transitions). Often, this change of mode does not occur frequently, and there is typically a gap of at least several seconds between two consecutive changes. This makes it possible to be selected by the user.

Relatively few papers are dedicated to high-level control. For most research purposes, the focus is on a certain mode of operation, and the experiments take place in controlled lab settings and are based on well-defined scenarios. However, reliable high-level control is crucial for the usability of exoskeletons for people in real-world situations and everyday life, where a variety of movements and gaits in different environments and terrain types are required and short transition times are necessary.

The inputs to the high-level controllers can come from the user (via input devices and/or sensors), the environment, or a combination of both. The output is usually a mode of operation. Artificial intelligence and machine learning methods are being increasingly used as a substitution for the user choice. The main motivation is to make the operation more automatic for the user, and possibly faster than manual input. Fundamental criteria for the usability of such methods are the real-time operation and short processing times, since decisions need to be made fairly quickly to allow enough reaction time for the lower-level controllers. Existing high-level control strategies are discussed in more detail below.

#### Explicit/manual user input (MUI)

The user directly determines the mode of operation of the exoskeleton, using input devices such as buttons [20–34] or voice commands [35, 36]. These methods are currently the most common due to their ease of implementation, higher predictability, and lower risk of errors. However, these advantages come at the cost of additional participation required from the user, which makes the user experience less natural, increases the cognitive load, and can slow down the operation. Moreover, this method is also prone to human errors which are more likely to happen during demanding tasks, long operation times, or with novice/distracted users. In this case, the challenge is both to make the user interface easy to use to minimize the learning time and the risk of manipulation errors, and also quick to use to avoid losing time in transitions. This is not trivial since the interface has to be used in a standing position, and the hands often have to hold crutches at the same time.

The explicit user input is commonly used in full-mobilization exoskeletons for complete spinal cord injury (SCI) patients, because no input can be obtained from the legs. It is also the most predictable for the user, which is important for trusting the device. In this case, buttons on the crutch handle, or a special wristwatch can be used. Voice command is not common because it requires speaking, which may feel awkward in public spaces. It is also more error-prone in noisy environments.

#### Brain-computer interface (BCI)

The user’s brain activity is measured using electrodes, amplified and analyzed to determine the mode of operation [37–39]. Among the different brain signal recording methods, currently electroencephalography (EEG) is predominantly used since it is non-invasive and therefore safer and easier to use. Despite the promising features of these methods, there are many practical challenges associated with them, including high levels of concentration required from the user (and therefore limiting simultaneous cognitive activities such as speech), artifacts with muscular activation (EEG signals at the surface of the scalp have an amplitude close to 100 μV [40], while electromyography (EMG) signals are several millivolts), rather lengthy procedures for electrodes placement, the need of training for the user and the algorithm, and being very slow (in the order of seconds) or limited to very few commands [39, 41–44]. A thorough review of brain-computer interfaces BCIs for lower-limb gait assistance devices in general can be found in [45], and an in-depth review of methods based on EEG in [46].

#### Movements recognition (MOV)

This type of controller changes the behavior automatically depending on how the user moves or is intending to move. The main advantage of this method is that it does not require any cognitive load or direct input from the user, making the interaction more intuitive and natural. For this method, generally joint sensors and IMU data (often from the upper body in persons with paraplegia) are processed by a machine learning or fuzzy logic algorithm to recognize the situation [47–64], although simpler threshold-based methods have also been proposed [65]. Sometimes, other types of signals such as the ground reaction forces or electromyography (EMG) are also used to infer the movement or the intention of the user [66–71]. Capacitive electromyography was also investigated [53]. In practice, often additional inputs are also required to complement these controllers (e.g. to disable them when the user needs to perform other activities while standing still in the device) since the movements of the user are not always sufficient to correctly determine the intention. In [72], the discrimination between walking and jumping is performed with a threshold on the phase difference between the two legs (shank segment), computed with the angle-speed diagram. Moreover, standing is detected if the magnitude of the phase vectors for the two legs is below a certain threshold.

#### Terrain identification (TER)

Generally, the most decisive factor in determining the mode of operation and high-level behavior of gait assistance devices is the terrain. Information about the terrain can hence be used to construct a high-level controller for such devices. In these controllers, embedded sensors are used to recognize the terrain type or obstacles in front of the user, in order to plan the steps accordingly [73].^2^ Sensors used for these high-level controllers are most often cameras (either usual visible-light cameras [74, 75] or 3D depth-sensing [41, 76–82]), but other sensors such as infrared distance sensors [83] or fusion of laser distance sensors and inertial measurement unit (IMU) [73, 84, 85] have also been utilized.

Terrain identification has recently gained attention in the fields of orthotics and prosthetics, and the body of literature exploring it is relatively small. Even the existing papers are limited to proof of concept implementations, demonstrating the performance of terrain identification algorithms without actually integrating them into the high-level controller of a device [73, 75, 79, 80, 83–85]. These techniques are usually computationally expensive because of the image or point-cloud processing. However, promising results have been demonstrated and with the advances in pattern recognition and machine learning methods, successful implementations of such controllers are to be expected in future research.

### Mid-level control

The mid-level is defined here as the continuous behavior of the robot, which computes the joints target torque or position, at each timestep of the main control loop. The mid-level controller plays the most important role in shaping the interaction of the device with the user, and the majority of the research on the control of exoskeletons is dedicated to this level. Although the output of the high-level controller also affects the behavior, it often only changes some parameters of the mid-level controller without fundamentally altering the essence of the interaction with the user.

In the proposed classification, the mid-level control blocks have been separated in two sublayers. As shown in Fig. 2, the “detection/synchronization” sublayer estimates the gait phase or gait state, which is a piece of information commonly needed by the “action” sublayer that actually computes the motor command. The first sublayer uses external inputs (from sensors and/or user interface) to determine the continuous phase or discrete state of gait. In the second sublayer, the desired physical output of the device is decided.

An exoskeleton controller can have a different control scheme for each joint. This is for example the case in [19], in which a simple spring is used for the ankle, an active damper for the knee, and torque control on the hip joint. Another example in [86] is an adaptive-frequency oscillator AFO-based impedance control for the hip, fixed position or zero torque control for the knee (depending on stance/swing), and event-triggered torque sequence for the ankle.

#### Detection/synchronization sublayer

The desired outcome of this sublayer is either the accurate gait phase (0–100%), or the gait state. Gait states are generally subphases of the gait cycle (e.g. stance/swing or finer divisions such as loading response/foot-flat/push-off), the kind and the number of which depend on each controller.

*Manual trigger by user (MAN)* This lets the user explicitly trigger the movement. This block is usually followed by the “Linear increase of the gait phase” and “Position profile”. This method is simple and used frequently to trigger the steps of a full mobilization exoskeleton. The trigger is generally a button ([20, 24, 26–28, 31, 87, 88]), but steps can also be triggered by EEG [42], although very slowly. It is worth mentioning that controllers in which the user manually triggers the start and stop of locomotion (and not the individual steps) such as [89, 90] do not belong in this category.

*Impose the movement (IMP)* Instead of synchronizing to the user, the robot imposes the movement continuously. So, it is the user’s responsibility to stay synchronized with the robot. This is sometimes the case with early-stage full-mobilization exoskeletons that test the continuous gait without providing a user interface to use them in real-use conditions [91–95]. Other common cases are brain-computer interface (BCI)-controlled exoskeletons that do not need crutches, with start and stop commands instead of having to trigger each step [41, 43, 44]. As opposed to the rest of the blocks, this one does not represent an actual function in the controller, nor does it have an output for its following block. Rather, this block is only used to emphasize the lack of synchronization. It is always followed by “Simple linear increase of the gait phase”, which then usually feeds the “Position profile” or “Torque profile” blocks.

*Event trigger (EVT)* This method can be found in many exoskeletons for partial assistance and full mobilization. It consists in using an event of the gait to start a step, a torque profile or to transition a state machine. The most common event is the heel strike, detected with a foot switch at the heel or (rarely) with an instrumented treadmill [96–107]. If the pressure sensor is located under the forefoot, the late stance can be detected instead of the heel strike [108]. The reference instant can also be recognized with an inertial measurement unit (IMU) on the shank, when crossing the zero angular speed [109]. A variant is to detect the point of “negative-to-positive power” of the ankle by looking at the ankle speed (one IMU on the foot, one IMU on the shank) [110], or with a classifier [111]. An alternative is to use an inertial measurement unit (IMU) in the foot sole [112–114]. Similarly, it is possible to detect the lift-off [48, 115]. A set of thresholds on the “analog” ground reaction force can also be used to discriminate several phases in the gait cycle [116–118].

Events in the kinematics can also be used. The peak value of the hip angle is used in [119–124], or similarly the peak ankle dorsiflexion angle [125]. In [126] the state machine is transitioned with thresholds on the knee angle and velocity. In [10], there is a threshold on the time-derivative of the pressure of the passive pneumatic actuator, which relates to the joint speed. In [127], a hidden Markov model is used to detect the gait phases from trunk and segment angles measured with an inertial measurement unit (IMU).

For full-mobilization exoskeletons, the steps can be triggered by weight shifting measured by the load cells under the feet [21, 128–130], by leaning toward the front or on the sides which is measured by the inertial measurement unit (IMU) [30, 128, 131], with a combination of the crutches load cells and the feet load cells [32], or a combination of the trunk tilt and the feet load [29].

*Adaptive frequency oscillators (AFO)* AFOs are dynamical systems with an oscillatory behavior that are capable of learning the features of a periodic input signal [132]. Due to the periodic nature of the gait, they can be used to determine the gait frequency and the phase. They can adapt quickly to a change of cadence, and do not need any prior knowledge on the shape of the gait pattern, except the fact that it is periodic. This makes them robust and makes the controller suitable for almost any user without the need for extensive parameter tuning or gait pre-recording. AFOs are usually fed with joints angles, but can also be used with any other periodic signal, such as the muscular activity, estimated using capacitive sensing [133] or interaction forces between the device and the user [134, 135].

AFOs can produce several useful pieces of information: the current progress in the gait cycle (0-100%), the frequency, and a filtered version of the input signal with no lag. Actually, the whole trajectory over the full gait cycle is modeled by the adaptive-frequency oscillator (AFO). These can be used in further action blocks, typically “Torque profile”, or “Impedance control”. The output has occasionally been directly used as a position reference as well [134, 135].

While AFOs are able to compute precisely the frequency and the joint angle value function over the gait cycle, the reference moment (usually the heel strike at 0%) is unknown so the absolute gait cycle progress cannot be determined. Several techniques exist to solve this issue:

- Foot switches can measure the instant of the heel strike [51, 136, 137]. This method is accurate, needs no heuristics, but requires an additional sensor. An inertial measurement unit (IMU) can also be used instead [138].
- A special feature in the joint trajectory (e.g. minimum or maximum value, or maximum slope) at a known gait phase can also be recognized, but this is subject-dependant and less reliable [122] (and probably [50]).
- Instead of a sine wave as the first harmonic, a known average human gait trajectory can be used [139–141]. This is less accurate if the user is walking in a non-typical way. Such an oscillator is called “PSAO” (particularly shaped adaptive oscillator) by the development team of the GEMS exoskeleton [139].
- Finally, strategies that do not use the absolute gait cycle progress can be selected, so that there is no need to obtain this information. This is the case for force fields that attract the joint towards its predicted position [56, 86], or compensation from a physical model (weight, inertia) [142].^3^ Note that “attracting toward the predicted position” is equivalent to using an impedance controller with the AFO-identified movement with a time offset (to follow the future) as the reference.

In [143], AFOs are also used, but the reference determination method is not explained. The Honda Stride Management Assist is also using a special AFO method according to a patent [144], but the details are not clearly documented.

The AFOs strategy is limited to the partial assistance paradigm, since the user needs to be able to initiate the gait and maintain it at least for a few steps.

*Simple linear increase of the gait phase (LNP)* This is the simplest way to determine or impose the gait phase. It consists of increasing linearly the gait phase over time, knowing in advance the step duration. If the movement is imposed all the time (IMP), the gait phase is looping continuously [38, 90, 91, 145]. If triggered manually [26, 35, 87] or with an event such as foot contact with the ground [97, 101, 146], lateral weight shifting [89], tilting the trunk [30, 147], or muscle activation (sensed via electromyography (EMG)) [82], it only runs once per trigger. The output of this block then feeds a position or torque profile.

*Time-interpolated gait phase (TBP)* This is the same as LNP, except that the gait cycle duration is determined automatically from the duration of the previous steps. This is very accurate if the gait is periodic and with a small inter-step variability. This method is very common for partial assistance [16, 96, 98–100, 102, 103, 105, 106, 108, 110, 113–115, 121, 123, 125, 148–155]. An extension of this method is to use a Gaussian probability density to reject outliers [156].

**Fig. 4:**
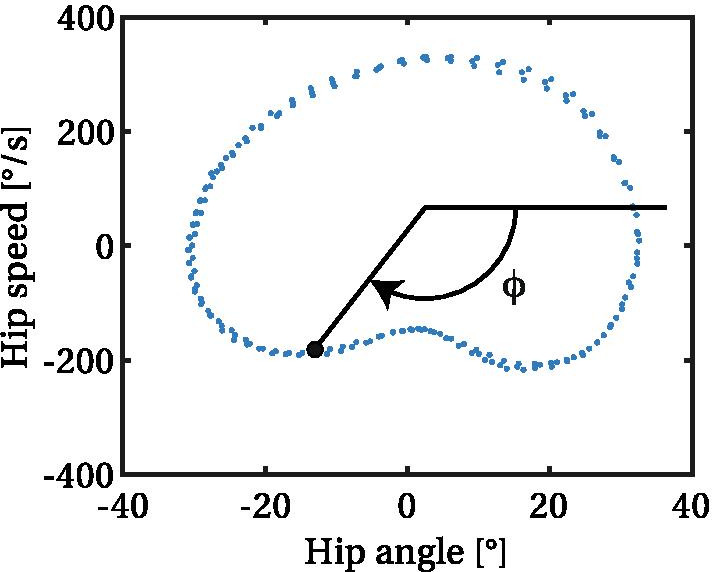
Example of angle-speed phase diagram. The data plotted is the hip angle during a few gait cycles of a test session with the exoskeleton SPRIINT (see [325])

*Angle-speed plot phase (ASP)* This technique consists in determining the gait phase from the angle and speed of a single joint. Intuitively, the function that maps a joint angle to the gait phase is surjective but not injective, because there are at least two solutions, due to the back-and-forth movement. So at best, if the joint trajectory is not bouncing, there are two possible gait phases for a given joint angle. However, the speed has the opposite sign for the way back, so it gives enough information to disambiguate the gait phase. In practice, these states are plotted on an angle-speed graph, and the phase angle can be extracted (Fig. 4). The center of the trajectory must be defined by prior calibration. The main advantage of this method is that it keeps its accuracy even if the gait cadence changes rapidly. However, it is very sensitive to bouncing, and is inaccurate if a joint moves little during part of the gait cycle. This is why it is not used with the knee joint. This method is used in [72, 157].

*Machine learning phase (MLP)* The gait phase can also be estimated using machine learning, with techniques such as support vector machine (SVM) or neural networks. The machine learning methods are diverse and complex, so they will not be explained here. All the found references used a different machine learning method and different inputs. A neural network fed with the trunk IMU data and hip encoder angles is used in [158].

An online Gaussian process regression is fed with the joints angles and interaction forces with the thigh cuffs in [59]. In [159], the gait phase is estimated with a decision tree, from the segments IMU data and the feet loads. In [160], deep learning is used on the shank and thigh IMU data and feet loads. In [111], a SVM is used with the shank IMU data. In [53], a quadratic discriminant analysis allows to get the gait phase from capacitive sensors measuring the thigh muscles contraction. Finally, in [161], a computer vision classifier can estimate the gait phase from the data of depth cameras located on the crutches.

*Other gait phase estimator (OTP)* The gait phase can also be estimated by other less common methods. One of the controllers proposed in [162] (“State Estimation” controller in the paper) estimates the gait phase by fitting the recorded joint angles and the foot loads to a reference model, using least-square regression based on the method from [163]. In addition to this method, another variant is also suggested for comparison in [163], which determines the gait phase based on minimizing the squared error between the instantaneous ankle angle and contact forces at toe and heel with those of a reference model (the first method is called “cross-correlation” and the second “k-nearest neighbors” in the original paper). However, the estimated gait phases have not been used in a controller, but have only been compared to evaluate the estimation accuracy.

*State machine (FSM)* Controllers can switch behavior depending on transitions triggered by events. This may be useful because some states of the gait are non-continuous. The best example is the foot contact, which is binary (swing/stance) and changes the dynamics of the leg. Many controllers use a state machine and different criteria have been utilized for transitioning between the states.

Most commonly, the ground contact state of the feet, or equivalently the ground reaction force (GRF), is used either for the entire foot to only distinguish between stance/swing [164–169] or considering local components (e.g. at the heel and under the toes) to further differentiate between stance subphases [48, 67, 86, 117, 148, 162, 163, 170–174]. The gait state can also be determined by computing the center of pressure (CoP) position of the stance leg with four load cells per foot, then applying a threshold to identify four states [175, 176]. In one paper, the subphases of stance were detected only based on the total ground reaction force (GRF) [116]. For some state machines, the ground contact status has been used as the only factor for transitioning the states [67, 117, 162–166, 171, 172], but it has also been used in combination with joint angle(s) [116, 167, 170], joint angular velocities [173], segment angles and angular velocities [48, 168], or the relative position of the feet [177]. In [148], the linear acceleration of the shank is also used in addition to ground contact data to improve the accuracy of heel-strike detection. The amount of time elapsed since the onset of swing has also been used in addition to ground reaction force (GRF) data to further detect subphases of swing [169].

Joint angles and angular velocities have also been used without the ground contact information to transition states [126, 178–182]. In [47], in addition to the angle and angular velocity of the knee joint, the moment at the joint and the angular velocity of the leg are involved in state transitioning. The authors in [19] have augmented joint angles with the forces and moments sensed in the exoskeleton segments to transition the state machine. In an alternative method, the difference between left and right joint angles (hip and knee) are used along with zero-crossing events of hip angular velocity to transition between the states [151, 152].

In [10], thresholds on the derivative of the pneumatic actuator pressure (which indicates the direction of movement intended by the user) are used for the transitioning. Surface electromyography (EMG) has also been used as another indicator of user’s intention to transition the states [66]. In [89], the estimated projection of the center of mass (CoM) on the ground relative to the feet is mostly used to transition between the states, but direct user input (via buttons) is required for transitioning in and out of the initial and final states, while transitioning between others (e.g. between shifting the weight to the stance leg and swing of the opposite leg) is initiated automatically.

Different states may only change the parameters and/or inputs to a controller (for example [48, 89, 117, 172, 183, 184]) or change the control strategy completely (for example [19, 61, 66, 86, 173, 185, 185]). It is also worth mentioning that sometimes the state machine does not involve any electronics, and is implemented using mechanical components only [164, 178, 179].

#### Action sublayer

The goal of this second sublayer is to generate a motor command, that can either be kinematic (angle or speed), or kinetic (torque or force).

*Position profile (PPR)* The goal of the position profile is to assist the user to move according to a predefined trajectory, supposed to be the intended one. The trajectories can be described in joint space or Cartesian space, often called “foot locus” for this second case. These trajectories are usually completely predefined based on recorded gait data from healthy people [48, 89, 91, 145, 186–188]. Databases of recorded trajectories from different healthy people have also been used in some strategies, where the controller chooses which trajectory to use depending on the situation [67]. In another approach, the trajectories have been recorded as a therapist manually guided the subject’s legs to achieve a desired gait pattern [187]. In [189], recorded trajectories from each subject walking in the exoskeleton in passive mode are averaged and used as reference. Some small modifications are generally necessary to account for user-specific and device-specific differences before actually using the trajectories recorded from healthy people for patients.

In many cases, the trajectories are significantly changed or fully generated at runtime, and some papers are completely dedicated to the problem of optimization/generation of trajectories [190–193]. In some studies, model-based computations [194–197] or polynomial minimum jerk trajectory generation methods [94] have been used to generate the trajectories offline. Trajectories can be generated so as to reach a certain target position/orientation in task space as well [191, 198]. For simpler implementations, the trajectory may also be defined approximately by a final target angle and a speed limitation instead of the complete path, and has been used for pneumatically actuated exoskeletons [88, 174].

However, the trajectories are not necessarily fixed or predefined. Online modifications can be applied to the baseline trajectories, as is the case in [199] and for the hip trajectories in [20] and [89] (only abduction/adduction angle in the latter). In [181], the user is free to move the legs during stance, and the baseline swing trajectory (from healthy subjects) is adapted at every step to match the leg configuration at the end of stance. More advanced methods have recently been proposed to automatically adapt the recorded gait trajectories from healthy people to the environment, and generate new trajectories for different types of terrain [82].

The trajectories could also be generated online, for example synthetic and parametrized trajectories can be used to adapt the foot clearance, step length and duration, peak joint flexion, etc. [77, 200]. The authors in [130] have proposed to generate the leg movement online to match the step length measured by a walker which is moved manually by the subject. In [192], a method is proposed to calculate the joint trajectories as a function of the movement of the crutches by the user’s arms, based on synergies extracted from the data of healthy subjects walking with crutches. Some controllers that are based on AFOs predict the joint trajectories online based on the estimated gait frequency and phase [190], and the future positions could be used as the reference for the actual joint [13, 56, 142]. Phase information estimated by AFOs has also been used to generate a custom trajectory in order to approximately achieve the desired power output [137]. In a different approach, the trajectory is generated online before each step based on the spring-loaded inverted pendulum (SLIP) model, taking the dimensions of possible obstacles into account [201]. For exoskeletons targeted at hemiplegic people, the movement of the nonparetic side at each step has also been recorded and used as the reference trajectory for the paretic leg [202, 203]. In a similar approach, kernel-based nonlinear filters have been used to learn the movements of the nonparetic leg as a function of gait phase online, and the learned functions are then used to generate the reference trajectory for the paretic leg [204].

Using position profiles is often associated with rigid position control in the full mobilization case. Then, the position profile is simply played back over time [27, 32, 33, 131]. The challenge is then to generate a set of gait trajectories that are comfortable, stable and able to overcome obstacles. For partial assistance, it is associated with impedance control [13, 145, 182, 205–207]. These trajectories can be played back over time [89, 205], or may be time-invariant (a tunnel or force field around the nominal path) [17, 181, 197, 208–211]. In [137], the reference profile is artificially generated and tuned to achieve a certain pattern of assistance. A combination of rigid trajectory tracking for some degrees of freedom and partial assistance around a trajectory for others has also been used [196].

The major drawback of the fixed-position-profile-based methods is their lack of flexibility, especially in the case of full mobilization. Even with many of the online modified or generated trajectories, the user is still forced to walk with the given gait pattern, which may not be suitable, and the trajectories are often specific to a particular terrain. For the partial assistance paradigm, even though the user has the freedom to diverge from the profile, it is still imposed and the controller will try to push in that direction, which might not necessarily help the user.

##### Torque profile (TPR)

Using a torque profile is the most simple and common method for partial assistance. A torque profile can be played back over time when it is triggered by an event [48, 96–100, 104, 111, 115, 119, 121, 123, 125, 151, 154, 165]. As the timing is very important, the torque profile may have (possibly online) tunable delay at the beginning of the torque profile. The torque profile itself may change over time, and be optimized online [105]. The torque profile can be as simple as a square pulse [103]. In some studies, the torque profiles are fine-tuned offline based on subjective feedback from the users [136, 212] or previous measurements from the users [169]. In others, they are optimized online for metabolic cost reduction [105, 213, 214]. The gait phase can also be estimated continuously, so the torque is applied as a direct function of the phase, independently of the time [50, 122, 136, 138–140, 143, 215–217], or combined with other inputs [51, 137, 141].

Probably the simplest case is the constant extension torque profile applied to the knee joint, when the leg is in single stance [176].

*Impedance controller (ZCT)* Impedance control is a widely used method in rehabilitation robotics and many other fields where the mechanical interactions with the user and the environment are significant [218]. As already mentioned, this method is used mostly in partial assistance paradigms where the human limbs are considered as active elements. Impedance control is often implemented such that the user gets the assistance torque only in case of a large deviation from the intended movement. This is usually called “assist-as-needed” and is mainly used for rehabilitation training, since it is believed to induce more active participation from the user compared to constant assistance or full mobilization, thus improving the learning and recovery.

In practice, impedance control can be implemented as an M/K/B (inertia/stiffness/damping) based dynamical system relating joint angles to torques [47, 49, 89, 127, 137, 153, 210, 219–223]. Either a reference target trajectory is played back over time [38, 145, 206], or the target is fixed and changes (also the stiffness and damping) only when the gait state changes [137, 166, 172, 180, 224–226]. In both cases, the target trajectory is generally in joint-space.

Another type of implementation is to use a force field with the joint states (angle, speed, acceleration, etc.) as inputs [181, 204, 227, 228]. A variation of the force field is the flow field controller proposed by Martinez et al. [229], which can also use the “state” given by several joints, while applying torque only at one [168]. A combination of both the force field and the flow field is suggested by Jabbari Asl et al. [230]. Note that using a multi-dimensional force-field in foot-locus-space to assist the leg to follow a pre-defined trajectory (such as the strategy used in [211]) is time-invariant, and is not the same as playing back a reference trajectory, even if both are classified as impedance control.

The impedance controller is usually implemented in software by changing the motor torque depending on the position and movement of the joint, but it can also be implemented using mechanical elements only (see "Torque control"). In [231], a negative impedance is tuned to compensate that of the leg to make walking less demanding for the user, since less effort is required to generate the same movement of the legs.

Finally, another possible strategy is to “attract” the joint to its future position with a virtual stiffness field [86, 142]. The future position can be predicted by exploiting the periodicity of the gait. The trajectory is typically identified online with an adaptive-frequency oscillator (AFO). This is equivalent to impedance control with the time-shifted identified trajectory as a target.

*Muscles activity amplification (MYO)* A joint torque that depends directly on the measured muscular activity is simple and can be very effective, since it can detect the intention of the user before the movement starts. However, it is usually limited by the fact that electromyography (EMG) sensors are time-consuming to set up, the signal amplitude may change because of changes of skin conductivity and muscles fatigue, and that some muscles are not accessible with surface electrodes. In addition, this technique becomes even more difficult in case of neurologic impairment. In fact, the muscles may have a lower contraction which reduces the amplitude of the measured voltage and hence the signal-to-noise ratio (SNR). This method is simply not usable with people affected with complete paraplegia because there is no voluntary stimulation of the muscles. It also cannot help people affected with coordination troubles, which would just be amplified by the device.

In this method, generally the calculated torque is directly applied to the joint [96, 116, 232–234, 234–236], but in some papers the torque is fed to an admittance model to generate position commands for the low-level controller [237, 238]. In terms of the approach to calculating the intended torque from muscle activity, several variants can be distinguished:

- The amplification of independent muscles activities is typically implemented with one artificial muscle per biologic muscle [239]. Its advantage is that the co-contraction of the biologic muscles also produces co-contraction of the artificial muscles, which allows to amplify both the torque and stiffness of the muscles. This can also be implemented with a single muscle; however, in this case, the biologic co-contraction will make the orthosis produce net torque. This approach has also been used in ankle exoskeletons with only unidirectional actuation (e.g. plantarflexion assistance only) [239, 240]
- The differential amplification of muscular activity computes the assistive torque by computing the difference of the activations [241]. Co-contraction just results in less torque. However, it may be approximative because of the non-linear activity/torque relationship.
- A variant is to let one activation inhibit the other [242].
- Instead of assuming the joint torque proportional to the raw measured activation, an alternative is using a calibrated musculoskeletal model to compute the joints torques from the measured activation [243].

In some approaches, muscle activity amplification is guided by a gait phase estimation method, where the activity of a certain muscle causes assistance only during a specific period of the gait cycle [116, 233]. A thorough review of these techniques can be found in [244].

In [245], the EMG-torque relation is estimated online during swing using a physical model. In [133], capacitive sensing is used instead of electromyography (EMG).

*Direct joint torque estimation (JTE)* The biological joint torques required for performing a certain movement can be estimated approximately using a simplified model with several weighted segments, and then (completely or partially) applied with an exoskeleton. Such a method has been used to assist squatting [92, 246, 247] or stair ascent [248] assuming quasi-static movement (neglecting inertia terms and only compensating the weight). A similar approach has been used in [49] to assist gait in different terrains (level ground, stairs, ramp) in conjunction with other strategies. In [249] the authors have used inverse dynamics (4 sets of equations depending on the contact point(s) with the ground) to estimate the joints torque. Another method that does not rely on an accurate model, is using ground reaction forces, shank angle, and shank length [250, 251]. It has also been proposed to use a spring-loaded inverted pendulum (SLIP) model to estimate the required biological hip and knee torques [252]. The point foot approximation is made, and the controller requires hip/knee joints angles, ground reaction forces, and center of pressure (CoP) position obtained with an instrumented treadmill. Similarly, in [253] the required stance ankle torque to compensate the effect of gravity has been derived based on a simple 2-DoF compass gait model. A mass model and ground reaction forces are used in [254] to estimate the hip and knee torques during gait, but the exoskeleton is actually not actuated.

*Model-computed action to keep balance (BAL)* Some control strategies address the issue of balance during gait based on different mathematical models of walking. For full mobilization exoskeletons, provided they have enough actuated degree of freedoms (DoFs) and that the user does not interfere, walking controllers developed for humanoids have been used [20, 194, 255, 256]. In [89] hip abd/adduction trajectories during swing are adapted online to improve lateral balance based on the “extrapolated center of mass” concept. In another approach, the difference between model-computed and actual GRFs have been fed to an admittance model to update the predefined trajectories online [198]. In the partial assistance paradigm, Zha et al. [257] have developed a controller only assisting in case of loss of balance, which is detected based on a quantitative balance metric. A model-based assistive torque is then calculated as the weighted sum of gravity, Coriolis, and inertial terms with weights determined using fuzzy logic.

*Neuromuscular model (NMM)* A class of bio-inspired controllers attempt to mimic the human neuromuscular system, consisting of virtual neurons and muscles. These virtual muscles are mathematical models based on the Hill-type muscle model [258] that generate torques as a function of the activation signal and the current muscle states (which are in turn a function of joint angles and angular velocities). The torque applied to each joint is then obtained as the algebraic sum of the torques generated by the virtual muscles acting on that joint. Some of these controllers are based on the neuromuscular reflex model from Geyer and Herr [259]. This bio-inspired model works based on feedback loops, or “reflexes”, that receive joint position information, ground contact and virtual muscle lengths as inputs, and generate activation signals for the virtual muscles. This concept was initially proposed as a model that can reproduce gait patterns similar to the natural human gait.

The reflex model has often been used to control prostheses, but implementations can also be found in the exoskeleton literature. In some modified versions, the activation signals of the muscles generated based on the reflexes are augmented with central signals generated by AFOs [51, 260], although in [260] and similar studies [261, 262] only the reflex-based controller has been tested with subjects. The activation can also be a function of electromyography (EMG) signals measured from the user’s biological muscles [243, 263]. In addition to joint torques, the neuromuscular model has also been used to determine stiffness [264]. In another work, the use of a neuromuscular model (which is explained in [265] and is different from the one used by the rest of the papers) has been mentioned, although it is not clear how it affects the proposed controller [266].

The main advantage of the neuromuscular model method is that it does not require a predetermined trajectory, and therefore does not impose the motion on the user. It rather follows the movements of the limbs and adapts to them, while being able to reject external perturbations. However, to operate properly, many parameters need to be tuned which can make the tuning process lengthy. Automated optimization with simulation tools is efficient, but such a process is difficult to implement with the actual hardware and user. Moreover, this method by itself is not suitable for complete spinal cord injury SCI patients since the user needs to at least initiate walking.

The neuromuscular reflex model has also been used in simulations of other assistive controllers to model the behavior of the human limb [152, 267]. In these papers, the neuromuscular model is simulated in parallel with the controller, receiving the torques generated by the controller as input and producing joint angles and speeds, which are fed back to the controller.

*Body weight support (BWS)* Body weight support was initially proposed as an augmentation to gait rehabilitation training, using stationary over-treadmill suspension systems [268]. The same idea can also be implemented using wearable lower-limb devices. Instead of providing assistance at the joints to move the legs, the idea is to relieve the user from a part of his/her weight, by having the exoskeleton pushing the trunk upward [11, 12]. This mainly works for the knee joint, because in stance, the partial gravity compensation consists simply in applying an extension torque. Note that this method is different from model-based gravity compensation which calculates joint torques required to resist gravity (e.g. the “gravity compensation control approach” in [92]). The latter approach has been categorized as “Direct joint torque estimation” in this review.

*Direct joint control by the user (DJU)* The joint torque can be directly controlled by a user (the wearer of the device or an external person such as a physical therapist), but this requires high cognitive load and prior training. This method has rarely been used, an example being [269] in which the pressure supplied to an artificial pneumatic muscle is proportional to the press of a button, controlled by a physical therapist or by the wearer. The actuator is used in an ankle exoskeleton to provide plantar flexion torque. In this study, the therapists could learn to properly activate the device to provide effective assistance, but most of the subjects could not successfully do it over 2 sessions.

An equivalent method for position-control also exists [270]. In this case, a pole is linking each foot the ipsilateral hand, with a multi-axis force sensor. Using an admittance controller, the position-controlled joints move according to the interaction force exerted by the hands, so that the feet “follow” the hands.

*Other function of feet/joints instant states (FJI)* The instantaneous values of the sensors such as joint angle or ground reaction force can be provided as inputs to a custom memory-less function, that directly computes joint torques [165, 234, 271] or positions [272–274]. Occasionally, electromyography (EMG) signals have also been used [275]. This information can also be supplemented with an estimate of the gait frequency [276]. Note that the type of functions used in this category does not fit into common strategies such as model-based torque estimations or virtual impedance functions. In [72], jumping is assisted at the ankle level by an impedance-like function producing an ankle torque proportional to the angular speed of the shank. In [116], the actuator pressure is proportional to the hip angle or the ground reaction force, depending on the current state (state machine triggered by a threshold on the ground reaction force value). In [277, 278] a passive mechanism using springs has been designed to compensate the gravitational forces such that the leg is approximately in static equilibrium in all configurations.

This method can also make the paretic leg follow the motion of the healthy limb in people with asymmetric pathologies [272], but this method is usable only if the movements of both legs should be symmetric, which is the case for sit/stand transitions (or jumping with joined feet) but not walking. A similar but more sophisticated method is estimating the desired trajectory of the paretic leg as a function of the instantaneous movements of the healthy side, based on inter-joint synergies derived from healthy gait [279–281].

*Other dynamical function of feet/joints instant states (FJD)* In a similar manner to the FJI category, although much less common, custom dynamical functions can also be used to calculate the desired action. In [282] the hip torque is computed as proportional to the difference of the sine of the hip angles, delayed by approximately 0.25 s. This makes the assistance torque adapt almost instantly to the variations in the gait cadence. In [227], gait-cycle-iterative corrections (as a function of the positioning errors in the previous steps) are applied to the baseline torque which is calculated using an impedance controller.

### Low-level control

This last layer is the closest to the actuators and therefore inevitably device-dependent. Most of the methods are not limited to exoskeletons but rather shared between many robotic applications, and the fact that they are being used in a gait assistance device does not affect the desired behavior (i.e. tracking of a reference input accurately while remaining stable). Therefore, papers focused only on low-level methods for exoskeletons and gait assistance devices are rare. Hence, we will limit this section to an overview of the existing methods and their relevant characteristics for gait assistance devices, without an exhaustive discussion about each method.

**Fig. 5:**
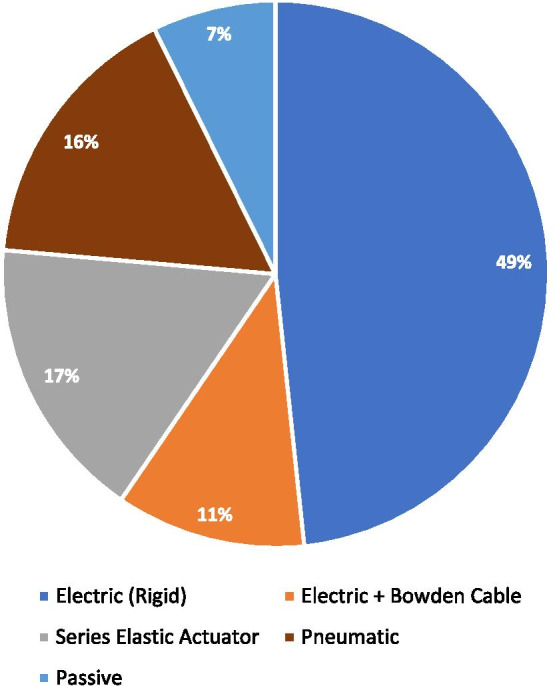
Distribution of actuator types in the reviewed articles. Studies in which the controller was not actually implemented in a real device or the actuator type was not mentioned were excluded for this analysis

Actuators used in robotics are generally direct-current motors that are current-driven, and the field of wearable robotics is no exception. This current regulation is performed by a high-frequency (typically $\ge {10}\;{\text {kHz}}$) inner control loop. The target current is determined depending on the type of low-level controller. The motor then transmits its torque to the load via a transmission system. Traditionally, rigid transmission systems such as gearboxes, ball screws, and belt drives were most prevalent, but introducing compliant elements into the transmission is becoming increasingly common in the applications involving interaction and force control. This added compliance improves the safety of interaction and the fidelity of force control. Bowden cables are also frequently used in exoskeletons, since they allow the transmission of forces over longer distances, making it possible to place the actuators more proximally or even off-board to decrease the burden of added inertia on the user. Devices with off-board actuators (also known as tethered) have been proposed as research test benches to compare the effectiveness of different control strategies independently of the device [283, 284]. Another category of compliant actuators frequently used in exoskeletons is pneumatic actuators, most often in the form of artificial muscles, which offer advantages such as low weight (neglecting the weight of the off-board compressor) and desirable passive properties. Finally, some assistive devices do not use any actuators but rather rely on passive elements that can store and release energy. Hybrid actuators have also been proposed, combining more than one actuator type per joint [285]. The distribution of the actuator types in the reviewed articles is shown in Fig. 5. For a detailed review of the different actuation technologies and particularly the compliant ones, the reader is referred to [286, 287].

While actuators with compliant properties are often used in partial assistance devices, actuators with rigid transmission are still the standard in full mobilization exoskeletons. Accordingly, in the low-level control, full mobilization exoskeletons use position controllers but partial assistance devices mostly rely on force/torque control schemes. In our classification of low-level controllers, we make a general distinction between position or speed controllers against torque controllers. The torque control category is then further divided into different methods.

#### Position/speed controller (POS)

The rigid position control is usually performed with a proportional-integral-derivative (PID) regulator. As most actuators have a large gear ratio and significant damping, the position control is usually straightforward. More advanced techniques exist [288–292], although such high positioning accuracy is generally not required for exoskeletons, because the structure is often slightly flexible and makes the legs movement less precise anyway. Moreover, relying on highly precise movements is not practical when there is some level of variability in the environment and the user can also affect the movement (e.g. using the upper-body) in unpredictable ways. Some of the more advanced controllers have focused on adding more compliant behavior to the position controller, such as the so-called “proxy-based sliding mode controller” [189, 293, 294], which offers smooth and gradual recovery in case of large errors. An iterative (over several gait cycles) online optimization of the torque profile to get the desired joint trajectory is presented in [227].

#### Torque control

The torque control is more challenging, because it requires a high bandwidth. A review of many low-level torque controllers can be found in [295]. For the case they tested (regular gait on a treadmill, Bowden cable transmission), they found that a proportional-derivative (PD) controller with iterative learning compensation was the best performing.

*Open-loop feedforward torque control (OLT)* Open-loop torque control is often chosen because it requires no torque sensor, which makes the hardware simpler. Two ways exist for its implementation. The first way is to set the motor current using a model of the actuator, including rotor inertia, dry friction, and damping [122]. This method is intended for stiff exoskeletons. Unfortunately, the inertia is hard to cancel because the acceleration is estimated from the position, which amplifies the measurement noise. The friction is also difficult to compensate because of its complex modeling. The second way, suitable for soft exosuits, is to run position control with a model of the stiffness of the system [112, 113, 143]. In [296], an admittance controller (force-to-speed) is followed by a speed control loop, to control the force. However, the cable-driven soft exosuit has a behavior that is too non-linear to get a consistent performance with the closed-loop control only. A feedforward component is then added, using a model that includes the suit stiffness, the actuator dynamics, and a thigh motion model (hip angle to cable retraction).

*Fast closed-loop torque control (CLT)* The closed-loop torque control is the classical way of controlling an accurate torque. It requires a torque sensor for feedback. The motor can be rigidly coupled to the joint (possibly through gears) or via a spring. The latter is called a series elastic actuator (SEA) and trades off some tracking bandwidth to get a better perturbation rejection performance. In other words, the softer the spring, the higher the torque regulation capability (a larger movement is necessary to achieve the torque perturbation), but the lower the ability to change torque fast (the motor has to spin more to achieve the torque variation). An advanced method to control the torque of a series elastic actuator (SEA) can be found in [297].

##### Gait-cycle iterative torque control (ITT)

Instead of controlling directly the joint torque with conventional fast closed-loop control, a position or speed sequence can be played back with a compliant actuator, which can do approximate torque control based on the actuator’s force-length relationship. This is well suited to systems that are soft and difficult to control. At the end of each step of the gait, corrections are made according to the comparison between the achieved and desired torques. Since the reaction time is one step, this is only accurate if the gait is periodic and regular, which is typically the case on a treadmill. The displacement/torque relationship can be estimated before the experiment, and a constant motor speed control results in the desired force profile [113, 114]. In [101], position-control is used on a Bowden cable to follow a trajectory, which translates to a torque at the ankle as a result of the elasticity of the exosuit. The trajectory is manually adjusted online to get the right force profile. In [109], a fixed voltage profile is triggered some time after the heel strike. It is also possible to tune the target trajectory online to get the desired work or average positive power [16, 102, 137]. In [16], a target trajectory is tuned online to get the desired average torque. In [298], a speed profile is tuned online instead. In [112], the speed/position profile is tuned online over several gait cycles, to get the desired torque profile.

*Special passive mechanical properties (PME)* It is possible to exploit the passive mechanical properties of the actuator, to benefit from some control properties that would require a larger and more complex actuator to emulate them with conventional force control, or additional sensors. A first example are the pneumatic actuators, that are compliant and with a limited tracking bandwidth. This is exploited in [234], where the “bang-bang” pressure controller does not result in a square torque profile, because of the smoothing by the limited actuator dynamics. In [226], the compliance of the locked actuator is used during stance. Actuation does not have to be bi-directional (e.g. pneumatic artificial muscles and Bowden cables can pull but not push), and this property is used to temporarily “disconnect” the actuator from the exoskeleton mechanically, to get a passive high-performance “transparency” (zero torque) without the need for a torque sensor [114]. In [26], the knee is position-controlled, but also features passive variable stiffness thanks to an additional actuator that controls the pre-tension of a spring. Achieving such compliance with a single actuator would not be possible, because of the high gear ratio and high inertia of the motor. A magnetorheological damper is used in [65] to vary the damping around the joint in different gait phases. The exoskeleton described in [72] uses a magnetorheological clutch, linked to a motor always running at full speed. This also makes it possible to mechanically “disconnect” the joint from the motor and get transparency, when the clutch is off. A similar system was presented in [299], but using a dual conventional clutch able to apply torque in both directions. In [300], the supply pressure of the pneumatic actuators is calculated so as to achieve a desired compliance (or equivalently, stiffness). In another study, a clutch is used to connect and disconnect a spring and a DC motor which is running during 85% of the gait cycle to stretch the spring, and is disengaged during the push-off period to let the spring release the stored energy and assist the ankle [178]. In a similar but simpler approach, a DC motor is used to compress a spring during stance (and this compression is also augmented by the dorsiflexion of the human ankle), and the stored energy is released at push-off [301]. Thus, using the spring as a passive element allows using a lighter motor with a lower power output.

##### Fully passive system (PAS)

A fully passive system does not use an actuator and relies solely on passive mechanical elements, such as springs and dampers. A small actuator may be present to control the state of the system, but will not exchange power with the joint [171, 173]. The net work of such systems can only be negative, but positive power can momentarily be provided if energy has been stored previously. The control behavior is more difficult to design and adjust, because of the mechanical changes required. However, passive mechanical elements have a very high power-to-weight ratio, and do not need a battery. Therefore, such exoskeletons can be significantly lighter to decrease the added effort and metabolic cost of carrying the weight of the device. The full actuator can be as simple as just a spring [213, 302–306] (hip joint only), [19] (ankle joint only). In [164], the spring is linked to a ratchet and a clutch to disconnect the spring from the ankle during the swing phase. A similar approach was described in [11]. In [171], the clutch is active, but the rest (a spring) is passive. In [19], the knee is linked to a damper via a clutch that is controllable from the software. A spring can also connect (indirectly) several joints together to perform power transfer [179, 307–309] (but this last one is an exotendon that does not really qualify as an exoskeleton). In [310], a rigid six-bar linkage with 1 degree of freedom (DoF) has been used to link the movements of knee and ankle joints and constrain the walking trajectory to that of a healthy person.

## Evaluation metrics

Evaluation metrics are necessary to assess the performance of an exoskeleton and compare it to others. As a human being is involved, they are unfortunately often inaccurate and not repeatable between subjects, and even between different trials with the same subject. A complete benchmarking scheme for bipedal locomotion was proposed by Torricelli et al. [311], summarizing many desired abilities, test cases, and metrics. This section briefly outlines the most common metrics that can be found in the literature to evaluate the exoskeleton-assisted gait. However, this is by no means an in-depth review of the evaluation metrics. For an in-depth review of the evaluation metrics, the reader is referred to Pinto-Fernandez et al.’s recently published review paper on this subject [9].

### Functional performance of the human-exoskeleton system

The performance can first be evaluated in terms of functional performance, which is the ability of the subject to complete a desired task. The scores obtained in Olympic sports (time to sprint 100m, maximum jumping height, etc.) are mainstream metrics but are not suitable for easier tasks such as walking. Other well-known methods are the 10-meter walk test (10MWT), the 6-min walk test (6MWT), the timed up and go (TUG) test [312], or the Fugl-Meyer assessment (FMA) [313]. These are often used with highly disabled patients and full mobilization exoskeletons [30, 314–316].

These metrics suffer from low repeatability, and the outcome depends on the subject’s motivation and effort as well.

### Metabolic cost

The metabolic cost is the amount of energy consumed by a subject to complete a task. These methods are useful because they capture the power exerted by the user, which relates closely to the required “effort”. However, the human body adapts slowly (with response times on the order of 1min [317]) and these methods are only usable for an exercise that lasts at least a few minutes.

*Heartbeat rate* Electrically-measured electrocardiography (ECG) or optical methods are used to measure this metric [122, 138, 315]. Electrocardiography (ECG) is usually preferred because of its better accuracy and robustness [318], although this distinction has recently been called into question [319]. The heartbeat rate measurement devices are easily wearable, compact and cheap.

*Gas exchange* Typically, the $O_2$ consumption is measured to estimate the metabolic rate [72, 303, 309, 320]. For these measurements, the exercise is performed continuously for 1–2 min until the steady-state value is reached. Another possible approach is to fit an exponential function [105] or a first-order function [214] on the transient part of the data.

### Muscular activity

There are various methods to approximately detect the level of activation of the muscles, which is another measure of the required effort from the user. However, these methods cannot measure the resulting joint torque, which is affected by other factors such as muscle fatigue, co-contraction of antagonist muscles, etc. These methods have a short response time, as opposed to the methods aiming to measure the metabolic cost.

*Electromyography* The most common method for monitoring muscular activity is the surface electromyography (EMG), with adhesive electrodes placed on the skin, over the muscles of interest [16, 321]. Implanted electromyography (EMG) sensors also exist to allow for monitoring internal muscles that are not accessible near the surface of the skin, but this technique is rarely used due to its invasive nature. electromyography (EMG) readings can be biased by the change of conductivity of the skin (as a result of sweating or migration of the conductive gel), and movement of the electrodes.

*Mechanomyography* In this method, muscular activation is measured by the change of volume or the vibration intensity of the muscles [322]. It typically gives the average muscle activity at a specific leg section.

### Joints mechanical power

By measuring the position of user’s body segments with a motion capture system, and the ground reaction forces with a force plate, it is possible to compute the movement and torque of each joint, and thereby its mechanical power. For this method, the knowledge of the user’s segment lengths and weights is needed as well. However, the joint mechanical power is not necessarily related to the muscle power [323].

## Discussion

The selected classification approach made it possible to describe 285 control strategies presented in 291 reviewed papers.^4^ A total of 31 blocks have been used. It should however be noted that the implementation details and the differences between various possible realizations of each block could be important and might even affect the performance outcome considerably. Comparing the performances was not intended in this review, and indeed it is practically impossible due to the differences in the target populations and tasks, testing procedures, and the reported outcomes in each study.

Selected controllers representing various possible combinations of the blocks are given in Table 2, along with their classification, actuator type, and a brief description of the control strategy. This table shows how the different blocks could be combined in numerous ways to form a controller.

**Table 2:** A selection of the reviewed controllers and their classification

References	High-level	Mid-level	Low-level	Actuator	Description
[170]	None	EVT-FSM-ZCT	CLT	SEA	From heel strike to mid-stance, the stiffness is incremented if foot slap is detected from GRF analysis. From mid-stance to toe off, zero impedance is applied to let the user perform powered plantarflexion. During swing, desired stiffness and damping are set based on the gait speed range
[332]	None	FJI	CLT	SEA	Required knee torque is estimated as the static torque resulting from the GRF, then it is applied with an amplification factor
[208]	None	PPR-ZCT	CLT	EM	A time-invariant tunnel is defined around a desired path, which is obtained from interpolation between the patient’s pre-training gait and that of a healthy subject. A virtual spring guides the leg back toward the tunnel when diverged. When inside the tunnel, an assisting force tangent to the path is applied
[30]	MUI	EVT-LNP-PPR	POS	EM	A watch is used to select the operation mode, then the fixed-trajectory steps are triggered with the trunk tilt
[241]	None	MYO	OLT	EM	Proportional EMG control; the applied torque is calculated based on the difference between flexor and extensor muscle activities
[103]	None	EVT-TBP-TPR	PME	PN	Uses an “algorithm” to predict stride time from heel switch data, then turns plantarflexion assistance on and off (applying constant pressure to pneumatic muscles) at pre-defined gait cycle percentages
[89]	None	EVT-LNP-PPR +BAL-ZCT	CLT	SEA	A state machine applies joint trajectories (fixed trajectories in the sagittal plane, online adaptation in the frontal plane based on XCoM to improve balance) and changes the impedances of the joints. Lateral weight-shifting triggers the steps
[44]	BCI	IMP-LNP-PPR	POS	EM	BCI control with 4 actions: “stand”, “walk”, “stop”, and “kick”. In one paradigm the subject triggers the walking and the steps are performed automatically. In another, the subject triggers each step
[117]	None	EVT-FSM-TPR	OLT	PN	State machine. Transitions using threshold on the feet pressure sensors (two per foot, one in front and one in back). Dorsiflexion torque applied at heel strike and toe-off, no assistance during foot flat, plantarflexion torque applied at heel off
[142]	None	AFO-ZCT?	CLT	SEA	AFO is used to estimate the gait frequency and joint angle, then the joint is attracted toward its predicted future position (equivalent to impedance control with the time-shifted, AFO-identified trajectory, as the target)
[150]	None	EVT-TBP-TPR +ZCT+BWS	OLT	EM	Torque sequence triggered by EMG. Also damping to limit the movement speed, and gravity compensation
[164]	None	EVT-FSM-ZCT	PAS	PA	A spring is only engaged during stance using clutch and ratchet mechanism (no electronics involved), to assist ankle plantarflexion
[256]	None	BAL	OLT	EM	Full mobilization with balance, resulting in crutch-less walking. Human and exoskeleton are considered as a single bipedal walker, and advanced control methods for bipedal robots are used. The details are out of scope for this review
[252]	None	JTE	CLT	EM	Estimates approximately the hip/knee torque using a spring-loaded inverted pendulum model, assuming point foot. Requires GRF and CoP position obtained from instrumented treadmill
[111]	None	MLP-ZCT+TPR	OLT	EM	Gait event detected with IMU and support vector machine: heel strike, heel off and toe off. Each event triggers a damping profile (HS) or torque profile (HO and TO)
[72]	MOV	ASP-TPR+IMP	PME	OT	Uses angle-speed diagram to get the phase. Discriminates between walking and jumping using the phase difference between the two legs. Walking: torque profile. Jumping: impedance control
[260]	None	EVT-FSM-NMM	CLT	SEA	Based on the reflex model by [259] which uses different reflex loops depending on stance/swing, but muscle activation signals also include another component simulating the input from central pattern generators (CPG) using adaptive-frequency oscillator (AFO). The CPG component has not been used in experimental tests
[41]	BCI+TER	IMP-LNP-PPR	POS	EM	BCI-controlled FSM decides 3 actions (turn left/right, walk front), and an obstacle detection system (3D camera + ultrasonic sensors) blocks the actions that result in hitting obstacles

Most of the references were chosen from the most cited papers (based on the number of citations in Google Scholar), and some were manually added to typify other possible combinations of blocks not covered among the most cited papers. Actuator abbreviations: EM: Electric Motor, OT: Other, PA: Passive, PN: Pneumatic, SEA: Series Elastic Actuator

In the high-level layer, the mode of operation of the device is determined, typically based on the type of gait or activity (e.g. normal walking, climbing stairs or, sit/stand transitioning). Currently few studies are addressing high-level control, mainly because in laboratory or clinical settings the mode of operation is often constant. In fact, only about 20% of the reviewed controllers addressed high-level control, many of which only briefly mentioned the method without focusing on it. 27 controllers were based on movement recognition, 15 based on explicit/manual user input, 13 based on terrain detection, and 5 based on brain-computer interfaces. 2 controllers combined brain-computer interfaces with terrain detection, and 1 controller combined terrain detection and movement recognition.

While high-level control can be ignored in applications such as rehabilitation, reliable high-level control is essential for devices that are ultimately intended to be used in everyday environments, particularly for full mobilization. Most commercial exoskeletons for full mobilization still rely on simple high-level control methods that require direct input from the user. Advanced methods such as terrain recognition and intent detection have recently received more attention, but most of the articles are in the preliminary testing and feasibility study phases. However, promising results have been demonstrated and these methods can be expected to be implemented in more devices in the near future.

The mid-level layer dictates the continuous behavior of the device in each operation mode (or in general, if there is only one mode of operation). This layer is central to the performance of assistive devices and consequently, the existing studies in exoskeleton literature are predominantly focused on this part. This layer is also usually more heavily affected by the fact that the controller is intended for an exoskeleton, whereas many of the high- and low-level controllers could be directly applied to other kinds of devices as well (e.g. prostheses and wheeled robots).

Mid-level control is further divided into two parts: (1) detection/synchronization and (2) action. While most control strategies found in the literature include both sublayers, some controllers operate directly on the raw sensory data (e.g. joint angles) and the synchronization is implicit (e.g. in muscle activity amplification) or nonexistent (the user has to synchronize with the device). Control strategies used in some papers consist of more than one set of functional blocks. Different sets of blocks could either be used simultaneously to complement each other, or separately based on the gait state or ambulation mode (switched by the high-level controller or a state machine in the mid-level layer itself).

Out of the 285 reviewed control strategies, 265 addressed mid-level control. The number of possible strategies in the mid-level layer is much higher than the others, with more than 40 possible variants identified in the reviewed articles, and many articles using more than one strategy.

In the synchronization level, 98 papers used event trigger, 35 imposed the timing, and 11 used manual trigger. Following these triggers, 72 papers used a simple linear increase of the gait phase and 52 used finite-state machines to switch between discrete gait states, while 31 carried out time-based interpolation to calculate the gait phase. For direct gait phase estimation, 26 papers used adaptive frequency oscillators, 9 used machine learning, 2 used angle-speed plot phase and 2 used other gait phase estimation methods.

In the action sublayer, impedance control was used 85 times, torque profile 64 times, trajectory-based position control 51 times, myoelectric amplification 22 times, function of foot/joint instant states 19 times, direct joint torque estimation 14 times, model-computed movement to keep balance and neuromuscular model 8 times each, and finally dynamical function of foot/joint states, bodyweight support and direct joint control by the user were used 2 times each.

The low-level layer is responsible for carrying out the desired “action” determined in the mid-level layer. The type of descending command (position or force/torque) from the mid-level layer determines the nature of the low-level controller. Typically the low-level control strategy can be a simple proportional-integral-derivative (PID) position regulator in full mobilization exoskeletons, or a torque controller (either open- or closed-loop) for partial assistance. Besides these methods which are very common in robotics, alternative methods also exist that can be applied to control the torque in actuators with mechanical compliance. Furthermore, simple forms of torque control have also been realized using only passive elements through storing and releasing energy. Clearly, the choice of the low-level control strategy is heavily influenced by the hardware of the device, which is in turn affected by trade-offs between wearability (e.g. weight and volume) and performance (e.g. power output and bandwidth).

249 out of the 285 reviewed control strategies included low-level controllers, although in some cases the type was not clearly mentioned. 60 control strategies were based on position control and 186 based on torque control. Among the torque-controlled devices, 57 had an open-loop torque controller, 55 a closed-loop torque controller, 36 used special passive mechanical properties, 18 were passive devices and 16 used gait-cycle iterative torque control.

**Fig. 6:**
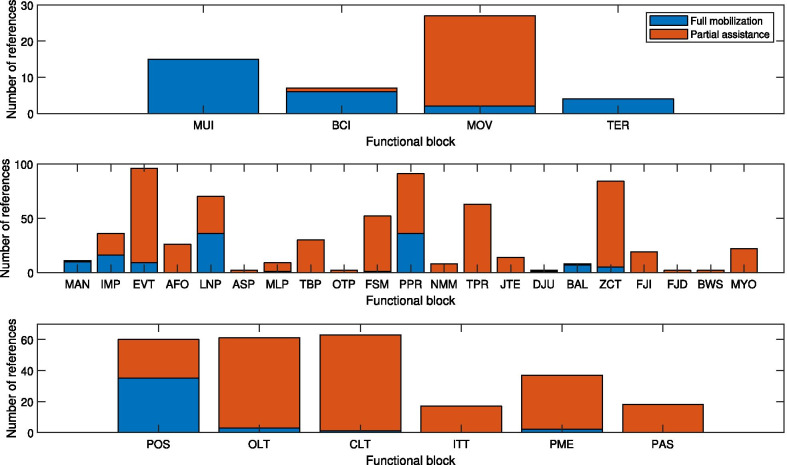
Number of references for each functional block (top: high-level, middle: mid-level, bottom: low-level)

**Fig. 7:**
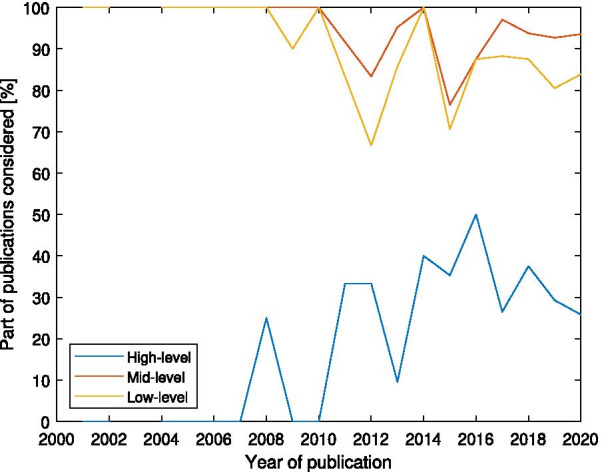
Percentage of the considered publications that addressed high/mid/low level, per year of publication

Figures 3 and 6 show that some control blocks are more popular than the others. For the high-level, it can be seen that in absolute terms, the high-level control method is not addressed often in the literature, although it is increasing over time (Fig. 7). The popularity of the manual user input (MUI) can be explained by the fact that the full mobilization exoskeletons are responsible for the full movements of the limbs, which should be adapted to many situations, and MUI is the most reliable and practical method today in this regard. For partial assistance, the movement recognition is more common because MUI and brain-computer interface (BCI) are not fast or convenient enough to use (the users have less impairments and higher expectations for usability), and the terrain detection is still in an early stage of development. For middle level, the leading blocks for partial assistance are event-trigger, impedance control and torque profile, probably because these are simple to understand and implement, and are able to cope with simple sensors (encoders, switches, etc.). The majority of the full mobilization exoskeletons use an imposed position profile (linear increase of the gait phase (LNP) + position profile (PPR)). For low-level, almost all the full mobilization devices use a position profile, because it is easy to implement with traditional actuators with high gear ratio, and because the mid-level associated blocks are mostly designed around reference kinematics, instead of joint torques or GRFs. For partial assistance, torque control is preferred, and open-loop (OLT) and closed-loop torque control (CLT) are the most common, because they are the traditional ways of torque control in robotic applications. Although CLT should be the most versatile (it does not require tuning to a specific movement, as opposed to exploiting special passive mechanical properties (PME) and gait-cycle iterative torque control), accurate and high-bandwidth torque control, it requires torque sensing and a careful design. OLT is simpler but less accurate, and less stable over time (e.g. due to changes in friction with temperature and wear). Generally, the most popular techniques are on one hand usable on simple hardware, so not requiring delicate sensing (e.g. muscles activity amplification) or special mechanics (e.g. PME, or even a fully passive actuator), and on the other hand easy to understand and implement. Typically, machine learning and neuro-muscular models are harder to implement because of the complex algorithms involved and the need for training data, so they are rarely used out of a simulated environment. It is remarkable that apart from the blocks related to imposing a position profile (continuously imposed movement, linear increase of the gait phase, position profile), the blocks are used almost only for full mobilization, or only for partial assistance, but there is little sharing. Manual user input, BCI, terrain detection, manual trigger, model-computed movement to keep balance, and position control can be associated to full mobilization, whereas movement recognition, event-trigger, adaptive-frequency oscillators, angle-speed plot phase, machine learning, time-based gait phase interpolation, other gait phase estimators, state machine, neuro-muscular model, torque profile, direct joint torque estimation, direct joint control by the user, model-computed movement to keep balance, impedance control, function of feet/joints instant states, dynamical function of feet/joints states, bodyweight support, muscles activity amplification, open-loop torque control, closed-loop torque control, gait-cycle iterative torque control, special passive mechanical properties, and fully passive system are associated to partial assistance.

Although objectively comparing the performance of the controllers is not possible (different target users, different tasks, different metrics, etc.), ten successful publications have been selected here, and have been used to highlight effective control features. In the context of the full mobilization, the four best performers to the CYBATHLON 2016 [324] have been selected [27, 28, 30, 90], because they proved that their system is fast and can overcome many obstacles. They are all using manual user input (MAN), because this is the most reliable method today. BCI is slow, limited to a small set of commands, and requires focus. Terrain detection is currently limited to over-simplified obstacles scenarios, but has a high potential because it will benefit from the current development of humanoids. Movement recognition is rarely used because the user has not enough voluntary control of the legs. For the lower layers, pre-defined gait trajectories with position-control (LNP+PPR+POS) are successful in practise, because they are simple to implement and reliable, and it is possible to tune them for different types of gait and obstacles. This imposed gait pattern can be triggered manually with the hands (MAN) or from the body movements (EVT). The exoskeleton in [195] is also remarkable because it is the first one truly able of hands-free dynamic walking. This new control technique, based on the balance control of humanoids (BAL) is promising, although less reliable (failure of the device cannot be recovered by the user, since there are no crutches) and currently slower. In the context of partial assistance, three passive [164, 307, 309] and two active controllers [123, 141] have been selected because they had the greatest results in lowering the metabolic cost of walking or running. The high-level control layer is rarely considered because most studies focus on steady-state walking or running. However, the transition between these two can be performed automatically using movements recognition (e.g. [123]). The most successful powered exoskeletons implement a torque profile, which can be made very efficient if the torque profile is tuned to the user. The synchronisation is done with an event trigger [123] or AFO [141]. There is no clear trend for the low-level torque control. Simpler, fully passive exosuits could also successfully break the “metabolic cost barrier” (as expressed in [7]), mostly with a variant of impedance control [164, 307, 309]. While the control can bring less power to the user, the ability to implement it on a dramatically lighter equipment makes the assistance outcome (assistance minus burden) beneficial.

Several full mobilization exoskeletons are already commercialized and the existing devices are reasonably successful in assisting people with paraplegia or severe lower-limb weakness. However, there still exists considerable room for improvement in their control strategies, particularly in the areas of balance, terrain adaptability, and walking speed. These potential areas of improvement are generally addressed in high-level control and the “action” sublayer of mid-level control. Recent preliminary studies in terrain detection methods have also demonstrated successful results, paving the way for more applied research on integrating these methods into actual exoskeletons. Investigating more generalizable online adaptive position profiles to decrease the reliance on fixed trajectories and terrain types also deserves more attention. For “crutched” exoskeletons, however, since the behavior of the device would become potentially unpredictable for the user, an advanced feedback system should be designed so that the crutches can be moved according to the expected movement of the legs. Finally, ensuring the safety and robustness of more sophisticated control strategies would be a major challenge in the transition from the laboratory to everyday use, because of the dramatically increased complexity. To achieve crutchless dynamic walking, recent advancements utilizing methods from the field of humanoid robotics have proven promising, and more progress in this direction is to be expected in the near future. Control strategies that effectively address fall prevention and recovery from tripping could also substantially improve the safety of full mobilization exoskeletons.

In the field of partial assistance, many encouraging results have been achieved as well, especially concerning metabolic cost reduction which is arguably the most sought-after target in this field [7]. Several studies have investigated the effects of different factors such as magnitude [99, 113] and timing [102, 141] of assistive torques, power delivery [102], and adaptation of the subjects [115, 121] on the metabolic cost reduction. This has led to a better understanding of the methods for effectively reducing the metabolic cost, and challenges such as compensatory behaviors in unassisted joints [16, 115]. Contrary to full mobilization in which further improvements mostly can come from the “action” sublayer of mid-level control, for partial assistance the performance of the “detect/sync” sublayer is equally important. In many partial assistance exoskeletons, the user has to bear the full weight of the device. This can be a serious hindrance, especially for people with existing weaknesses. Therefore, improving the design of partial assistance devices to reduce their weight could be as important as improving the control strategy. Controllers that can work with simpler hardware are at an advantage. In the same vein, soft exoskeletons (exosuits) have received increased attention from the researchers and this trend is likely to continue.

For future research, more comparative studies testing different control strategies on the same hardware and in similar conditions could be valuable. The developments in machine learning methods have successfully been utilized in detection of the environment, locomotion mode, and also the gait phase. But these methods have not been integrated with subsequent action blocks frequently. In the future, controllers with machine-learning-based environment/activity recognition and synchronization can become more common. Robust and reliable detection of the terrain or the activity mode using machine learning can make exoskeletons more autonomous and much easier to use in everyday situations. Accurate detection of the gait phase can on the other hand improve the effectiveness of the assistance provided by the exoskeletons. For the latter purpose, however, simpler methods can also provide sufficient accuracy.

**Fig. 8:**
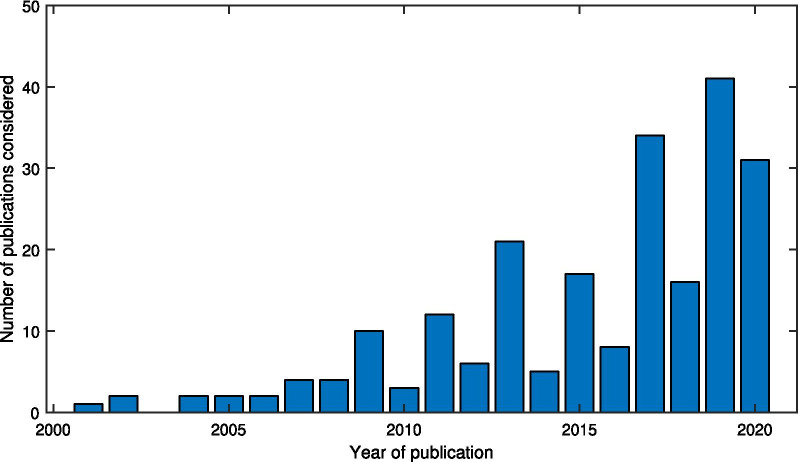
Number of reference considered, per year of publication

With a large scope, not limited to a specific application or type of device, this review was intended to provide an organized overview, to help the new researchers in the field understand the vast range of control strategies for lower-limb exoskeletons to assist the gait. The proposed layers and blocks structure is well suited to sort and compare the existing controllers, and probably the future ones. Such organization work is essential because the topic of exoskeleton control tends to accelerate its expansion (Fig. 8), which makes understanding the existing methods increasingly difficult. However, it is not a practical tool for the inverse process, which is the synthesis of new controllers. In fact, the classification is made so that it is independent of the target application or device, but these are important for the design of a performant controller. For example, some blocks are irrelevant for the full mobilization of complete spinal cord injury SCI users (e.g. body weight support and muscles activity amplification), and some implementations require specific sensing which may not be available on the actual hardware (e.g. electromyography (EMG) sensors or foot pressure sensors). Also, this classification gives no guidance on how to choose the implementation of each selected block, because the performance of each implementation is not compared. In addition, there is no guarantee that the complete controller results in a useful behavior. Hence, choosing a path through the blocks is a complex task. Finally, the proposed blocks set emphasizes on the *processing* that leads to the desired behavior, but does not show the *sources* of information. For example, it is not visible that a controller uses the movement of the sound leg to control the paretic leg, or that another uses the foot contact information to produce torque at the hip. This can be considered as a limitation because intention detection is a key challenge for these controllers, and sources of information play a decisive role in intention detection. The main reason is that in the literature, the techniques are designed for and tested with an actual device with fixed inputs (e.g. sensors locations), but these techniques may still be applicable to other exoskeleton topologies.

## Conclusion

In this paper, an overview of the existing literature on the control of lower-limb orthotic devices for gait assistance was provided, plus a brief overview of the metrics commonly used to evaluate the performance of the controllers. An effort was made to focus on the core concepts used in each controller, and to separate them from different possible implementation methodologies and hardware platforms, whenever possible. A 3-layer hierarchical structure was proposed for the classification of the controllers, conceptually similar to the suggested framework in a previous review article [4].

The different possible control approaches in each layer were then represented by atomic functional units in the form of blocks. While most of the blocks could be implemented using various methods, the overall function remains largely the same. This type of classification and decomposition facilitates the comparison of the different existing approaches in terms of control by abstracting out the basic idea regardless of the implementation details. It allows capturing not only the differences but also the similarities among different approaches.

A vast number of methods were identified showing considerable heterogeneity, each one being tailored to a specific kind of application, target population, and performance objective. No comparison was made among these methods in terms of effectiveness and performance outcome, mainly because a general comparison would be pointless when the ultimate objectives and target populations are fundamentally different. Furthermore, even among the studies sharing these features, the protocols used for testing and the reported performance metrics (if any) often do not match and thus there is not enough information to make systematic comparisons. Regardless, it can be stated that many significant improvements in terms of performance outcomes have been achieved recently, as pointed out by other recent reviews as well [1, 7].

Currently, the most successful full-mobilization exoskeletons are controlled with manually selected modes (MUI), setting pre-defined trajectories that are manually (MAN) or automatically (EVT) triggered. The main limitations are that the crutches prevent the use of the hands for other tasks, and switching from one mode to another is time-consuming. To address these issues, there are promising developments ongoing on dynamical balance (BAL) and terrain awareness (TER), both supported by the recent advances in powerful, compact, low-power, embedded computers. Nevertheless, these devices will be more complex, bulky and expensive than the current crutched exoskeletons. Humans are already aware of the terrain and know what movement to perform, they are just unable to command the exoskeleton accurately and quickly enough. Consequently, more research work on efficient user interfaces could improve the current generation of exoskeletons and make them quicker in less structured environments (single step stair, short sideways slope, speed bump, etc.).

For partial assistance, the best results have been obtained with event-triggered (EVT+TPR) or AFO-synchronised (AFO+TPR) torque profile, possibly tuned to each user with human-in-the-loop optimization, and torque control. These techniques are exploiting the periodicity of the gait to keep the torque pattern synchronized with the legs. This is why it can be expected that they are less efficient in unstructured environments, where the gait pattern is less regular. State-less techniques (such as FJI) could solve this issue but they have not been addressed often in the literature. Hence they may deserve more research effort.

These improvements in controllers, along with other advancements in technology, hardware and design have taken gait assistance exoskeletons one step closer to becoming mainstream, although many challenges still need to be resolved before making the move from laboratories to real-world usage. This would call for the research on exoskeleton control to start moving toward more comprehensive studies with more realistic scenarios and protocols in the near future. Such studies necessitate more interdisciplinary collaborations among control researchers and specialists from various other disciplines, from physiologists and clinicians to design engineers and conformity assessment bodies for medical device regulations in different countries and regions.

## Supplementary Information

**Additional file 1.** The full data base of the reviewed records and their classification according to the proposed framework.

## Data Availability

All data generated or analysed during this study are included in this published article and Additional file 1.

## Footnotes

1 Although for severely disabled patients, full mobilization is also used in the early stages of rehabilitation, most rehabilitation devices fit in the partial assistance category better.

2 Many of the papers cited in this section are taken from prosthetics literature, but the fact that the terrain identification systems have been designed with a prosthesis in mind does not affect the outcome and all of the results are equally applicable for orthoses as well.

3 This second variant should technically not be part of this review, because it was proposed for an upper-limb exoskeleton, even though the paper also addressed lower-limb assistance with another controller. It could probably be applicable to lower-limbs also, but no publication could be found with this principle.

4 Some of the reviewed papers included more than one controller, and some papers —by the same research group— used the exact same controller several times, therefore the number of control approaches does not exactly match the number of papers.
